# Platinum Mine Workers’ Exposure to Dust Particles Emitted at Mine Waste Rock Crusher Plants in Limpopo, South Africa

**DOI:** 10.3390/ijerph17020655

**Published:** 2020-01-19

**Authors:** Maasago M. Sepadi, Martha Chadyiwa, Vusumuzi Nkosi

**Affiliations:** 1 Department of Environmental Health, Faculty of Health Sciences, University of Johannesburg, Cnr Sherwell and Beit Street, John Orr Building, 7th Floor, Doornfontein Campus, Doornfontein, Johannesburg 2094, South Africa; mchadyiwa@uj.ac.za (M.C.); vusi.nkosi@mrc.ac.za (V.N.); 2 Environment and Health Research Unit, South African Medical Research Council, Johannesburg 2094, South Africa; 3 School of Health Systems and Public Health, Faculty of Health Sciences, University of Pretoria, Pretoria 001, South Africa

**Keywords:** South Africa, platinum mining, crusher plants, dust, inhalable, respirable, risk assessment

## Abstract

The South African mining industry is one of the largest producers of platinum (Pt) in the world. Workers in this industry are exposed to significant amounts of dust, and this dust consists of particles sizes that can penetrate deep inside the respiratory region. A cross-sectional study was conducted to evaluate dust exposure risk at two Pt mine waste rock crusher plants (Facility A and B) in Limpopo, South Africa. Workers’ demographic and occupational information was collected through a structured questionnaire, a walk-through observation on facilities’ processes, and static dust sampling for the collection of inhalable and respirable dust particles using the National Institute for Occupational Safety and Health (NIOH) 7602 and the Methods for Determination of Hazardous Substance (MDHS) 14/4 as guidelines. Only 79% of Pt mine workers, used their respiratory protective equipment (RPE), sixty-five percent were exposed to work shifts exceeding the recommended eight hours and 8.8% had been employed for more than ten years. The mean time-weighted average (TWA) dust concentrations between Facility A and B showed a significant difference (*p* < 0.026). The Pt mine’s inhalable concentrations (range 0.03–2.2 mg/m^3^) were higher than the respirable concentrations (range 0.02–0.7 mg/m^3^), however were all below the respective international and local occupational exposure limits (OELs). The Pt mine’s respirable crystalline silica (SiO_2_) quartz levels were all found below the detectable limit (<0.01 mg/m^3^). The Pt miners had increased health risks due to accumulated low levels of dust exposure and lack of usage of RPE. It is recommended that an improved dust control program be put in place which includes, but is not limited to, stockpile enclosures, tire stops with water sprays, and education on the importance of RPE usage.

## 1. Introduction

In response to the demand the mining industry has expanded by 3.7% in 2017, with platinum (Pt) comprising 26% of mineral exports [1]. The Pt industry became the major contributor to the South African (SA) mining sector after the decline of gold production [2]. According to the Chamber of Mines of SA (2018), the Pt industry generated 8 billion rand in sales in 2017 [3]. Furthermore, the SA Chamber of Mines indicated that more than 175,000 people were employed in the sector in 2018 [3].

Haque et al. (2014) defined Pt mining as the process of extracting the mineral from the Earth’s crust and the removal of the economic ore [4]. Pt mining categories include underground and opencast operations, with the latter presenting exposure of mine workers to dust particles [4]. Pt mining activities generate residues such as waste rock, which are materials that are not valuable economically. The mine waste generated during the course of Pt mining activities is regulated by the SA Mining Residues Regulations [5] published under National Environment Management Amendment Waste Act (NEMWA, Act 26) of 2014 [6]. The innovative methods currently used in managing Pt mine waste include crusher plants that turn underground mine waste rock into various construction products. The crusher plants perform opencast mining activities which are part of the growing small-scale mining sector [7]. However, these facilities’ mining processes involve crushing and screening of the mine waste rock, which inevitably produces dust particles. These dust particles are classified into coarse and fine particles and exposure occurs when these particles are inhaled during mining operations and pose various respiratory risks depending on the size of the particle [8].

There are major health challenges in the mining industry such as pulmonary tuberculosis (PTB). The other challenge is silicosis as most mineral rocks have crystalline free silica (also known as quartz). Surface Pt mining is a leading cause of exposure to excessive dust particles that are harmful to health and are associated with various causes of silicosis and pneumoconiosis [9], with the respirable crystalline silica (SiO_2_) quartz generated during stone crushing linked to increased occurrences of pulmonary tuberculosis and chronic lung disease. Ndaba’s (2017) study also found the existence of silicosis in Pt miners in 544 out of 6662 certified cases in 2004 [10]. This is attributed to exposure to dust particles, specifically silica dust, which is considered a risk factor for the development of PTB [3]. The Department of Mineral Resources reported 2838 TB cases in 2012, with the Pt mining sector contributing the second-highest number (895) [3]. Phillips et al. (2014) mentioned that workers with the disease or the potential to develop disease from silica dust exposure might be working in the Pt mining sector due to cross-recruitment, which often occurs from gold to Pt mines [11]. To explore the potential Pt risk in mine workers who had never had worked in another mining sector aside from Pt mining, Nelson and Murray (2013) conducted a descriptive case-series study in SA from 1975 to 2009, which showed an autopsy crude prevalence of 0.06% and 0.30% potential silicosis in the case of Pt miners [12].

Occupational health and safety (OHS) should remain the number one priority in mining. A research study showed that mining operations were still the leading cause of exposures harmful to health and associated with various causes of occupational accidents and diseases [7]. For example, in SA, Pt mining contributed 25 fatalities and injuries between January and October 2017, with similar statistics for 2016 within the same period [13]. Pt mineworkers’ exposure variations are due to the different mining activities, particle amounts, and the particle size’s ability to penetrate a specified respiratory region.

Pt mining in SA is under-researched, which hinders the publication of information in line with dust particles exposure and associated health effects. A study by Nelson and Murray (2013) indicated that most Pt miners’ medical records were not complete; thus, not enough evidence was available to make conclusive findings with regard to dust production and exposure to dust particles during Pt mining [12]. Another challenge was that the migration of miners from one mine to another makes it difficult to find conclusive evidence of dust particle exposure in Pt mining. To minimize the chance of including miners that had been exposed to dust particles outside of the Pt mining sector, Nelson and Murray (2013) conducted a descriptive case series study in SA from 1975 to 2009 [12]. This study included miners who had never worked in any mining sector other than the Pt industry. The results showed evidence of health effects as a result of exposure to dust particles, for example, silicosis in some of the miners. The probable health effects that may occur due to exposure to any amount of dust are determined by the chemical properties of such dust particles.

There are several standards to manage production of dust particles and exposure during Pt mining, but there is little evidence that these standards are sufficiently addressing the problem of dust production and the exposure of mine workers to this dust. Therefore, when protective occupational exposure limits (OELs) are set for mining, a large number of workers will be protected.

This study was conducted at two pre-selected mine waste rock crusher plants (named Facility A and B) situated in the Limpopo Province of SA, which is known as the Bushveld Igneous Complex. The methodologies used in this study were selected to confirm the dust particle exposure of Pt mine workers in the crusher plants by characterizing dust into size, mass concentration, and hazardous pollutants through exposure monitoring conducted over the standard eight-hour day using a time-weighted average (TWA), with a comparison made with what is acceptable in terms of local and international occupational health standards. The first stage was to identify and evaluate the workplaces (i.e., mine waste rock crusher plants) through a walk-through survey and followed by a self-administered questionnaire. The third stage was conducted using area (static or fixed) dust sampling in the chosen mine waste rock crusher plants.

## 2. Materials and Methods

### 2.1. Study Design and Sample

This is a descriptive cross-sectional study using quantitative measures to gather information on occupational dust particle exposure. A stratified sampling method was used with an inclusion criterion predefined by homogeneous exposure group or based on work-task, and the Pt mine workers’ were divided into occupations to establish the difference in exposure. Office workers and security guards were excluded as they were not involved in the production or handling of the waste rock. The target sample size to complete the questionnaire included the existing 100 permanent Pt mine workers. However, out of the 100 total Pt mine workers in the inclusion criteria of both facilities, only 34 respondents (34%) were reached for this study due to low production and facilities process schedules.

This methodological approach involved identifying of the characteristics of a population at one point in time and presenting the situation in the facilities as it is, in order to confirm or investigate the Pt mine workers’ dust exposure phenomenon through dust sampling.

### 2.2. Data Collection Instruments

In this study, data collection was divided into three stages, which included a walkthrough observation, a close ended self-administered questionnaire, and static dust sampling.

#### 2.2.1. Walk-Through Observations

The first stage was to identify and evaluate the workplaces’ (waste rock crusher plants) handling processes by means of a walk-through survey along with the site manager of each facility. This process was necessary to provide the basis for the quantitative dust assessment.

#### 2.2.2. Self-Administered Questionnaire

The second stage was conducted using a previously validated questionnaire (Table S1) from the British Medical Research Council. It was then developed with specificity to this study by the authors and the University of Johannesburg’s statistician using close-ended questions to acquire the Pt mine workers’ biographic and occupational details. The questionnaires were distributed to the 34 respondents on site during lunch breaks, and all participants accepted the English version and did not request copies translated to any other languages which, prior to the study, were planned to be made available upon request.

#### 2.2.3. Area Dust Sampling

The third stage entailed using area static dust sampling in the crusher plants conducted in October 2018, following the guidelines of the international standards of National Institute for Occupational Safety and Health (NIOH) 7602 [14] and the Methods for Determination of Hazardous Substance 14/4 (MDHS) [15] over eight working hours.

The nine work stations, (five in Facility A and four in Facility B) were based on the facilities’ scheduled process flow on the date of sampling, and contributed to the collected 18 dust samples (nine inhalable and nine respirable). The workstations were identified during the walk-through survey in the two waste rock crusher plants (Facility A and Facility B). In Facility A, five workstations were identified, namely: the feeder, screener, twister crusher, excavator, and front end loader (FEL). Facility B had four workstations, namely: the feeder station, screening station, multi-stages crusher station, and excavator.

The dust collection instruments used were multi-fraction Institute of Occupational Medicine (IOM) samplers, which concurrently collected inhalable and respirable dust particles and meets the international standards. The larger inhalable (<100 μm) dust particles were drawn through a filter paper, which was placed between a cassette and a support grid to trap dust particles. The smaller respirable (<10 μm) dust particles were sampled by a cyclone using the polyvinyl chloride filter enclosed in a cassette to separate smaller particles from larger ones.

The flow of the calibration method using a rota-meter was 2.2 Liters per minute for the multi-fraction IOM samplers [14,15], and was checked before and after every sampling to avoid errors in reporting. The sample filter cassettes were covered and stored in cases when transported to the sampling location. To ensure that the outcome of samples were traceable, information such as facility identity, sampling area, the sample identification, and pump start time and end time were recorded on an exposure assessment field sheet adopted from the Department of Minerals and Energy of SA [16]. The collected dust sample for this study is communicated as mass of dust per cubic meter (mg/m^3^) of air.

### 2.3. Statistical Analysis

A quantitative analysis method was followed for this research for analysis of the numerical data. Statistical analysis was conducted using Statistical Package for the Social Sciences (SPSS) version 25 [17] for all questionnaire data. Descriptive analyses such as frequencies, percentages, and means were used to summarize data as appropriate. Gravimetric analysis according to MDHS 14/4 was used for the dust samples collected. The analysis of this study further used Fourier Transform Infrared Spectroscopy with potassium bromide for analysis of silica particles [15]. The gravimetric method used the TWA dust concentration over an eight-hour work shift, which was calculated using the following formula [18]:
Sample volume (m^3^): Flow rate (1/min) × time (min)Correction filter mass (mg): Post filter mass − Pre filter massSample mass (mg): Post weight sample mass − Pre weight sample massCorrected sample (mg): Corrected sample mass − Correction factor.Concentration (mg/m^3^): Mass ÷ Volume (mg/m^3^).TWA dust concentration: Concentration × Total sample time (min)

The independent sample *t*-test was conducted with SPSS to determine if the TWA dust concentrations from the two facilities were significantly different, with a significance level considered at α = 0.05.

## 3. Results

### 3.1. Workers Demographic and Occupational Characteristics

The highest number of Pt mine workers was found at the cleaning activity group, at 20.6%, followed by the crushing activity group, at 17.6% (Table 1). Literature has proven that that all workers are at risk of developing health effects due to dust exposure; however, the risk level could differ per worker due to demographic characteristics such as age and gender [13,19]. The Safety in Mines Research Advisory Committee (2001) handbook explains the relationship between gender, age, personal protective equipment (PPE) usage, length of service, and the cumulative dust exposure as being closely related [19]. The results showed 85.3% males as compared to 14.7% females (Table 1).

Referring to this study’s demographic information, which could affect the significance of exposure, the Pt mine workers’ average age, was found to be 37 years (range 23 to 68 years). The largest age group was 30–39 years (41.2%) with similar numbers of workers in each facility. The smallest age group was found to be the 50–59 year olds (2.9%) which existed only in Facility B. The oldest individuals (above 60 years of age) were from Facility A alone (Table 1). Out of the 34 Pt mine workers, most of the workers (38.2%) had working experience of one to four years, while few Pt mine workers (5.9%) had worked ten years or more (Table 1).

There was inconsistency or inadequacy in PPE usage, with 3% of participants revealing that they were not being provided with PPE and, for those provided, 21% admitted to not using their PPE at all times (Table 2). It was found that the 66.7% of the Pt mine workers who admitted to not wearing PPE at all times were working for longer than the recommended eight-hour work shift (Table 2). Analysis of occupational characteristics such as duration of exposure by work shift (Table 2) and length of service (Table 1) showed that 65% of the participants from both facilities were working for longer than the recommended eight-hour shifts, and 8.8% of participants had performed ten or more years of service.

### 3.2. Dust Particle Concentration (Mass)

In SA, the OELs are published by the Department of Mineral Resources, under the Mining, Health, and Safety (MHS) Act of 1996 (Table 3) [20]. The Pt mine dust respirable particulates were compared to the SA’s MHS Act OELs, which are the only relevant OELs that exist for this particular hazard (Pt mine dust respirable particulates). Table 3 reflects high respirable TWA dust concentrations in Facility B when compared to Facility A. The highest levels of TWA respirable dusts for both facilities were found at the feeder stations (Facility A at 0.6 mg/m^3^ and Facility B at 0.7 mg/m^3^). The lowest levels of respirable TWA concentrations for each respective facility were found at FEL A (0.022 mg/m^3^) and excavator B (at 0.03 mg/m^3^).

Pt mine dust respirable particulates from the facilities were further characterized into Sio_2_ quartz. This characterization was important as probable health effects may occur due to exposure as determined by the chemical properties of such dust particles. The international OELs used for comparison purposes were from United States Department of Labor under the Occupational Safety and Health Administration (OSHA) as shown in Table 4 and Table 5 [21]. Other countries such as Australia, Belgium, Denmark, France, Greece, Sweden, and the United Kingdom have OELs for respirable SiO_2_ quartz set at the same amount as SA (0.1 mg/m^3^), and Italy and Finland have OELs similar to that of the OSHA OEL, which is set at 0.05 mg/m^3^. The respirable SiO_2_ quartz concentrations were found to be below the respective local and international OELs of 0.1 mg/m^3^ and 0.05 mg/m^3^ at all work stations (Table 4).

There are no set OELs for Pt mine dust’s inhalable particulates in both local and international organizations; hence, the OELs for inhalable particles not otherwise classified (PNOCs were used for this study. The highest inhalable TWA concentration in both Facilities were found at the Feeder stations (Facility A at 0.7 mg/m^3^ and Facility B at 2.2 mg/m^3^) and the lowest concentrations for each facility were found to be at the excavators (Facility A at 0.1 mg/m^3^ and Facility B at 0.03 mg/m^3^).

Feeder B showed the total highest inhalable TWA concentration (2.2 mg/m^3^) as demonstrated in Table 5. Table 5 further shows that the lowest TWA concentrations of inhalable dust were at excavator B (0.03 mg/m^3^).

Combining the results of Table 3 and Table 5, the mean time-weighted average (TWA) dust concentration for both inhalable and respirable dust particles was found to be 0.4 mg/m^3^ (range from a minimum of 0.2 mg/m^3^ to a maximum of 2.2 mg/mg^3^), with a standard deviation of 0.5 mg/mg^3^. The mean inhalable particle TWA concentration was found at 0.6 mg/m^3^ and respirable particle TWA concentration at 0.2 mg/m^3^ over an eight-hour work shift. There was a significant difference between the facilities (*p* < 0.026), showing Facility B to have levels 0.2 times higher than Facility A.

### 3.3. Workstation Risk Profiling

Although exposure assessment is an integral component of environmental epidemiology and OHS, risk profiling according to concentrations is vital in dust exposure assessments. Based on the TWA concentration values obtained during the exposure assessment phase, the SA-OELs, and the health effects of respirable and inhalable dust; the workstations were then risk-profiled (Table 6) according to the guidelines in Table S2. Pt mine respirable dust (<5% SiO_2_) is associated with pneumoconiosis, Pt mine respirable dust (>5% SiO_2_) is linked with silicosis, and inhalable mine dust is related to physical irritation. The Pt mine respirable SiO_2_ quartz concentrations were not subjected to a risk rating as all samples were found below the detectable limits. Out of 18 dust concentration results, the risk-analysis matrix obtained showed a very high risk level at two stations, namely, feeder A and crusher B for respirable dust, with the workstations of lowest risk being the screen, excavator, and FEL. However, the inhalable dust particulates values in all stations were found to be below 30 as per the classifications, showing that the risk of exposure to inhalable dust particles is acceptable or tolerable when compared to respirable dust particles.

## 4. Discussion

The risk of dust exposure from the mining industry depends on the specific activity, duration of exposure, characteristics of dust, and workers demographic characteristics. There was exposure to dust particles at all mine waste rock crusher facilities. These solid particles are classified as chemical hazards which Pt mine workers come into to contact with, through inhalation over an eight-hour work shift.

The majority of the Pt mine workers were found to be cleaners (20.6%), with four females and three males, which is not surprising as traditionally cleaning services have been dominated by women [22]. The crushing activity group was the second largest group, representing 17.6%.

Particle size and mass concentration are crucial factors for the characterization of dust. Most mining activities have greater numbers of coarse particles as compared to fine particles. The inhalable dust fraction TWA mean concentration (0.6 mg/m^3^) was higher than that of the respirable fractions (0.2 mg/m^3^). This study’s comparison of inhalable and respirable dust results is supported by a scientific research conducted on three open cast mines in India which also showed that inhalable particulate matter (PM) of 10 micrometers or more in diameter (>PM_10_) concentrations were between 22% and 36% higher than respirable fractions (<PM_10_) [23].

The OELs that apply to Pt mining have been set locally and internationally as a mitigating method with respect to dust particles in the workplace. However, there are still struggles with compliance. The findings from this study present TWA concentration levels that are much lower than the local and international inhalable and respirable dust exposure limits set between 15 mg/m^3^ and 3 mg/m^3^, which have been deemed unsafe by various studies. A relevant exposure response study conducted among gold workers in SA showed that the OEL set at 0.1 mg/m^3^ was not sufficient to protect the workers [22]. A scientific report on respirable dust concentration showed results of 0.018 mg/m^3^ to 0.035 mg/m^3^, with values lower than SA’s OEL of 0.1 mg/m^3^; however, the same results were higher than the American Conference of Governmental Industrial Hygienists’ limit of 0.025 mg/m^3^ [13,23]. Furthermore, the MHS report stated that 95% of exposure measurement should be below the Pt dust respirable particulate level of 1.5 mg/m^3^ [24].

OHS studies have also proven increased risk of exposure due to demographic and occupational characteristics such as age, gender, PPE usage, and duration of exposure by work shift and job service length [8,10,18]. The British Medical Association (2016) stated that there is “an accelerated decline in forced expiratory volume in one second (FEV1) and forced vital capacity (FVC) with age and that the respiratory system reaches maximal function between the ages of 20 to 27 years, thereafter lung function decreases progressively” [19]. Furthermore, the SA mining industry reported a mean age of 54 years for 19,531 pneumoconiosis cases between 2004 and 2012 [10]. Ndaba (2017) further reported specific Pt mining results that showed certified silicosis cases, with most of the affected miners being in the age group of 40 to 59 years and the age group with the lowest rate aged 30 to 39 years, with no cases found in individuals aged less than 30 years [10].

Comparing the findings of the present study to those of the British Medical Association (2016) and Ndaba (2017) [10,19], the Pt mine workers aged between 20 and 39 years (65%; *n* = 17 males and *n* = 5 females) wee a non-vulnerable group, whereas those aged between 40 and 68 years (35%; *n* = 12 males and no females) were a vulnerable group, with a more than 20 milliliter FEV1 annual decrease. The lower incidence amongst younger workers as compared to older workers is mostly due to the scientific statistics of cumulative exposures or latency periods, which indicates increased health risks among elderly workers [13]. The 36.8-year average indicates non-vulnerable Pt mine workers in terms of health risks, which is supported by a SA mining industry occupational disease study [10]. However, the presence of different age-groups in the facilities selected for this study indicates variety of the risk to health. The occupational characteristics such as duration of exposure by work shift and job service length showed that 65% of the participants from both facilities worked for more than the recommended eight hours, and that 8.8% of participants had performed 10 or more years of service which is an indication of an increased health risk.

The SA mining industry has recorded occupational lung diseases such as silicosis, occupational TB and workers pneumoconiosis as the key challenges of health. In terms of Pt mine health effects, a recent SA mining industry study pertaining to lung diseases amongst male and female miners found that 93% of the diagnoses of pneumoconiosis were in men and only 5% in females [10], which could pose potential threat to males more than females.

The results showed further lack of legislation compliance with regard to simple dust mitigation measures such as usage of PPE for an entire shift, even where management made such equipment available to workers. The lack of RPE usage and longer duration of exposure has been associated with the risk of pneumoconiosis; a scientific study found the risk of pneumoconiosis to be higher in people that had been exposed to mineral dusts for long periods of time and in cases of the inconsistent use of RPE [25]. The relationship between PPE usage and duration of work further shows that employees wearing PPE continuously for longer than a normal shift tend to find it uncomfortable. The respirator, based on laboratory-measured performance data, shows that the filtering face piece 2 (FFP) used in both facilities deals with moderate levels of fine dust and can be used during sanding, cement, drilling, and cutting. However, it must be noted that the use of FFP 3 is recommended in the mining sector, where silica could be present.

The risk-profiling matrix (Table S2) showed two high-risk profiled workstations with respirable dust (Table 4); this risk-rating evaluation indicated that measures should be implemented to reduce the potential harm at the highest-risk stations.

In terms of long-term exposure, the facilities’ waste rock particles may also go through chemical reactions during overtime storage, which may generate additional products that could be toxic human health [26].

It has been clearly shown in the literature for the past 40 years that static samplers (also called area samplers) are not adequate when used without personal sampling for characterizing worker exposure. However, they are excellent for determining the continuing adequacy of the process and process controls. That means that in order to protect the Pt mine workers there should be methods used for monitoring their health status.

## 5. Conclusions

The general conclusion that can be drawn from the present study is that Pt mine workers had increased health risks with accumulated low levels of dust exposure due to lack of RPE usage. No conclusions could be drawn on personal health due to the study not focusing on personal dust exposure or medical examinations but rather on determining risks related to processes or workstation exposure at each crusher plant.

Personal samples are generally higher in concentration when taken in the same area. The potential limitation in the study is that personal sampling results are universally considered more appropriate for the protection of workers. Therefore, further studies could extend the exposure monitoring by including medical surveillance and personal dust sampling in order to further establish the impacts on health of the concentrations found at Facility A and Facility B.

Pt mine workers in these crusher plants need to be protected from exposure through the use of advanced technologies that are more efficient. Some measures could include the use of bag-houses or collector dumps which can be placed at the machines stone deposition end, which discharge the products close to the ground and reduce discarding of dust. Other measures include the use of wet methods for stockpiles or road-dust haulage; tire stops with water sprays which reduce rollback underneath vehicles and suppress dust at the stockpile deposit areas; use of enclosed hopper dumps; limiting vehicle movements (such as clients’ collection trucks, or delivery trucks) during processing hours and installing speed limits that can reduce dust production; limiting the magnitude and duration of exposure through task rotations or rest periods for workers; provision of appropriate RPE approved by national and/or international standards [8]; and encouraging usage of RPE amongst all persons working in this sector. The most important measure is the training of employees to support a health and safety culture that promotes zero tolerance to dust exposure.

## Figures and Tables

**Table 1:** Distribution of platinum mine workers’ demographic and occupational characteristics by facility.

	Facility A		Facility B		Total	
	*N*	%	*N*	%	*n*	%
**Age Groups (Years)**						
Mean/average age	36.8	N/A	36.9	N/A	36.8	N/A
Maximum	68	N/A	58	N/A	68	N/A
Minimum	23	N/A	28	N/A	23	N/A
20–29	6	30	2	14.3	8	23.5
30–39	7	35	7	50	14	41.2
40–49	5	25	4	28.6	9	26.5
50–59	0	0	1	7.1	1	2.9
60 or more	2	10	0	0	2	5.9
**Total**	**20**	**100.0**	**14**	**100.0**	**34**	**100.0**
**Gender**						
Males	15	75	14	100	29	85.3
Females	5	25	0	0	5	14.7
**Total**	**20**	**100.0**	**14**	**100.0**	**34**	**100.0**
**Occupational (Activity) Groups**						
Crushing	4	25.0	2	14.3	6	17.6
Loading and offloading	3	10.0	2	14.3	5	14.7
Screening	2	10.0	2	14.3	4	11.8
Final storage	2	10.0	1	7.1	3	8.8
Transporting	1	5.0	1	7.1	2	5.9
Cleaning	3	15.0	4	29.0	7	20.6
Water sprayer	1	5.0	1	7.1	2	5.9
Diesel attendant	1	5.0	0	0.0	1	2.9
Supervising/foreman	1	5.0	1	7.1	2	5.9
Weighing bridge	1	5.0	0	0.0	1	2.9
Welding	1	5.0	0	0.0	1	2.9
**Total**	**20**	**100.0**	**14**	**100.0**	**34**	**100.0**
**Length of Employment**						
Less than 1 year	5	25.0	1	7.1	6	17.6
1 to 5 years	9	45.0	8	57.1	17	50.0
6 to 9 years	4	20.0	4	28.6	8	23.5
10 years or more	2	10.0	1	7.1	3	8.8
**Total**	**20**	**100.0**	**14**	**100.0**	**34**	**100.0**

%: percentages; *n* = number of samples; N/A = not applicable.

**Table 2:** Cross tabulation of personal protective equipment (PPE usage (with % within usage) and work shift (with % within each work shift) for each facility.

Facility	Personal Protective Equipment (PPE) Usage			Total
	Never Use	Almost Every Time	At All Times
A	1 (100.0%)	3 (50.0%	16 (59.3%	20 (58.8%)
B	0 (0.0%)	3 (50.0%)	11 (40.7%)	14 (41.2%)
**Total**	**1 (100.0%)**	**6 (100.0%)**	**27 (100.0%)**	**34 (100.0%)**
	**Work Shift**			
	**<8 hours**	**8 hours**	**>8 hours**	
A	1 (16.7%)	2 (33.3%)	17 (77.3%)	20 (58.8%)
B	5 (83.3%)	4 (66.7%)	5 (22.7%)	14 (41.2%)
**Total**	**6 (100.0%)**	**6 (100.0%)**	**22 (100.0%)**	**34 (100.0%)**

**Table 3:** Workstations’ Pt mine dust respirable particulate in comparison with the TWA OELs.

Workstation	Facility	Time-Weighted Average (TWA) Concentration (mg/m^3^)	OEL Comparison
Hazard: Pt Mine Dust Respirable Particulates (<5% Crystalline Silica Quartz) (<10 μm) OEL: 3.0 mg/m^3^ OEL Type: TWA-OEL Reference: MHS Act, 1996			
**Feeder station**	A	0.586	BL
	B	0.081	BL
**Screening station**	A	0.169	BL
	B	0.051	BL
**Crusher station**	A (twister)	0.432	BL
	B (multi-stages)	0.697	BL
**Excavator**	A	0.028	BL
	B	0.026	BL
**Front end loader**	A	0.022	BL

BL: Below limit. MHS: Mining, Health, and Safety; TWA: time-weighted average; OEL: occupational exposure limit.

**Table 4:** Respirable crystalline silica (SiO_2_) quartz OELs of workstations in comparison to other established OELs.

Workstation	Facility	Respirable Crystalline Silica (SiO_2_) Quartz (mg/m^3^)	OEL Comparison			
Hazard: Pt Mine Dust Respirable Particulates (>5% SiO_2_) (<10 μm)						
			MHS Act, 1996		OSHA (2016)	
			OEL: 0.1 mg/m^3^ OEL TYPE: TWA		OEL: 0.05 mg/m^3^ OEL TYPE: PELs	
**Feeder station**	A	<0.01	BL	BL	BL	BL
	B	<0.01				
**Screening station**	A	<0.01				
	B	<0.01				
**Crusher**	A (twister)	<0.01				
	B (multi-stages)	<0.01				
**Excavator**	A	<0.01				
	B	<0.01				
**Front end loader**	A	<0.01				

<0.01: Below detectable limit; PEL: Permissible exposure limit.

**Table 5:** TWA concentration OEL comparisons of Workstations’ inhalable particles not otherwise classified.

Workstation	Facility	TWA Concentration (mg/m^3^)	OEL Comparison			
Hazard: PNOCs Inhalable/Total Dust Particulates (<100 μm)						
			MHS Act, 1996		OSHA (2010)	
			OEL: 10 mg/m^3^ OEL TYPE: TWA		OEL: 15 mg/m^3^ OEL TYPE: PELs	
**Feeder station**	A	0.672	BL	BL	BL	BL
	B	2.172				
**Screening station**	A	0.402				
	B	0.069				
**Crusher station**	A (twister)	0.579				
	B (multi-stages)	0.904				
**Excavator**	A	0.132				
	B	0.029				
**Front end loader**	A	0.295				

BL: Below Limit; PEL: Permissible exposure limit.

**Table 6:** Workstation’s risk rating and classification (Table S2).

LEVEL OF RISK		Very High (AA)		400 and Above	
		High (A)		200 to 399	
		Moderate (B)		70 to 199	
		Low (C)		20 to 69	
		Tolerable (D)		<20	
Workstation		Probability of Exceeding the OEL (P)	Exposure (E)	Consequences (C)	Risk Rating (PXEXC)
**Health Hazard: Pt mine respirable dust**					
Feeder station	A	3	10	15	**450**
	B	0.5	10	15	**75**
Screening station	A	0.5	10	15	**75**
	B	0.5	10	15	**75**
Crusher station	A	1	10	15	**150**
	B	3	10	15	**450**
Excavator	A	0.5	10	15	**75**
	B	0.5	10	15	**75**
FEL	A	0.5	10	15	**75**
**Health Hazard: Pt mine inhalable dust**					
Feeder station	A	3	10	1	**30**
	B	6	10	1	**60**
Screening station	A	1	10	1	**10**
	B	0.5	10	1	**5**
Crusher station	A	3	10	1	**30**
	B	3	10	1	**30**
Excavator	A	0.5	10	1	**5**
	B	0.5	10	1	**5**
FEL	A	1	10	1	**10**

Red code: Very high risk level; Mustard code: High risk level; Yellow code: Moderate risk level; Green code: Low risk level; Lime code: tolerable risk level.

## Supplementary Materials

The following are available online at https://www.mdpi.com/1660-4601/17/2/655/s1, Table S1: Questionnaire. Table S2: Risk rating determination band table [18].
