# Supplementary material for: Platinum Mine Workers’ Exposure to Dust Particles Emitted at Mine Waste Rock Crusher Plants in Limpopo, South Africa
Source: Int J Environ Res Public Health. 2020 Jan 19;17(2):655. doi: 10.3390/ijerph17020655 (PMC7014327; doi:10.3390/ijerph17020655)
Supplement: Supplementary file 1 [file ijerph-17-00655-s001.pdf]

Supplementary material

Table S1. Questionnaire.

Instructions: For each question with numbered answers, use an X or tick in the box.

Thank you for your participation

SECTION A: BIOGRAPHICAL DETAILS

1. What is your gender?

Male	1
Female	2

2. How old are you?

- ……… years old

SECTION B: OCCUPATIONAL DETAILS

3. What is your job title/profession?

Machine operator	1
Residential engineer	2
Driver	3
General worker	4
Other, please specify: --	5

4. Which work task/site are you operating from? (Choose one answer)

Crushing	1
Grinding	2
Blasting	3
Loading and offloading	4
Screening	5
Transporting	6
Final storage	7
Other, please specify: --	8

5. How long have you been working on this site?

- ------------- Years
- ------------- Months
- ------------- Days

6. Approximately how many hours are you exposed per day?

Less than 8 hours	1
8 hours	2
More than 8 hours	3

7. Are you provided with protective clothing (PPE)?

No	0
Yes	1

8. Which type of PPE?

		No	Yes
8.1.	Not applicable	0	1
8.2.	Face shield	0	1
8.3.	Respirator	0	1
8.4.	Dust Mask	0	1
8.5.	Other, please specify: --	0	1

9. Do you wear or use your PPE?

Never use	1
Occasionally/sometimes	2
Almost every time	3
At all times	4

10. In your work task, how often do you change your PPE?

Not applicable	1
Never change	2
Daily	3
Weekly	4
Monthly	5
Quarterly	6
Annually	7
Other, please specify: ---	8

Table S2. Risk rating determination band table (SIMRAC 2001).

Risk factor		Value
**PROBABILITY OF TWA EXPOSURE> OEL** What is the level of exposure?	Exposure > OEL-c or exceeding the TWA-OEL more than threefold or mixture of exposure with an index of >3.	**10**
	Exposure of more than or equal to OEL-TWA of or equal to OEL-TWA or mixture of exposure with an index between 1 and 3.	**6**
	Exposure of more than or equal to 50% of the OEL and < OEL or mixture of exposure with an index between 0.5 and 1.	**3**
	Exposure of more than or equal to 25% of the OEL and < 50% of the OEL or mixture of exposure with an index between 0.25 and 0.5.	**1**
	Exposure of more than or equal to 10% of the OEL and < 25% of the OEL or mixture of exposure with an index between 0.1 and 0.25.	**0.5**
**EXPOSURE PERIOD** How many times people are exposed to the event and for how long	Continuous exposure for 8-hour shift or more.	**10**
	Continuous exposure for up to 6-hour shift - frequent, daily.	**6**
	Continuous exposure for up to 2-hour shift - often weekly.	**3**
	Short exposure periods, a few times per month.	**2**
	Unusual, at least once yearly.	**1**
	Rare.	**0.5**
**CONSEQUENCES OF EXPOSURE** Severity of harm or damage that could occur	One or more mortality from an occupational disease.	**100**
	Life threatening/disability injury or illness. Multiple occupational diseases cases. Miscarriage (very serious).	**50**
	Irreversible health effects of concern or serious illness. Compensable occupational disease. Possible miscarriage (serious).	**15**
	Reversible confirmed health effects. Occupational disease.	**7**
	Minor ill health.	**1**

**Calculated risk**	**Band**	**Risk classification**	**Action**
400 and above	AA	Very high risk	Consider discontinuation
200 to 399	A	High risk	Immediate action required
70 to 199	B	Moderate risk	Correction required
20 to 69	C	Low risk	Attention necessary
<20	D	Tolerable risk	Monitor

Red code: Very high risk level; Mustard code: High risk level; Yellow code: Moderate risk level; Green code: Low risk level; Lime code: tolerable risk level.
